# Influence of Mentorship and the Working Environment on English as a Foreign Language Teachers’ Research Productivity: The Mediation Role of Research Motivation and Self-Efficacy

**DOI:** 10.3389/fpsyg.2022.906932

**Published:** 2022-06-15

**Authors:** Yanping Li, Lawrence Jun Zhang

**Affiliations:** Faculty of Education and Social Work, The University of Auckland, Auckland, New Zealand

**Keywords:** research motivation, research self-efficacy, research productivity, mediation effects, university teachers, foreign language teachers

## Abstract

Research productivity is an important criterion for the university to assess teachers. Studies about factors that affect teachers’ research productivity are increasing nowadays. It is generally agreed that academics’ research productivity depends on how much mentorship is provided to them and how the current working environment is mediated by their research motivation and self-efficacy. Despite the increasing amount of the literature along this line, we know little about what kinds of situations that Chinese university English as a foreign language (EFL) teachers are in and how they regard the importance of mentorship and what roles their working environments would play in affecting their research productivity. To fill the research gap, we utilized the snowball method to collect the survey data from 546 Chinese EFL tertiary teachers. The results show that mentorship is not correlated with research productivity while the working environment has a positive direct correlation with it. Both motivation and self-efficacy mediate the working environment and research productivity significantly. Specifically, only extrinsic motivation has a negative mediation influence on teachers’ research productivity; teachers’ intrinsic motivation and self-efficacy play a positive mediation role in affecting their research productivity.

## Introduction

International and local university rankings have been a symbol of the university’s influence and competitiveness (Morze et al., 2022). Research productivity takes an important position for its weight in the world university ranking. For example, research takes about 40% of the total score in the 2022 Academic Rankings of World Universities. Similarly, in the Times Higher Education ranking system, research and citations (research impact) account for 30% respectively of the overall score in the 2022 rankings. As the core of science, the publications are significant for communication and exchanging current findings, knowledge, and ideas (Fox, 1992). Besides, teachers’ professional development relates to not only teaching but also their career advancement based on publications and other forms of research productivity (Borg and Liu, 2013). Additionally, Zhang (2021a) found that teachers felt the institutional push to do research, and are pressurized to get it published in international journals (Mu and Zhang, 2018). In their study, Zheng and Gao (2016) found that Chinese scholars preferred using Chinese and English in their effort to pursue research excellence by leveraging on languages they are proficient in for research and publications. This is particularly true of scholars, who work in language-related disciplines (e.g., language and literacy education, including foreign language acquisition). Research has actually been an important means to teachers’ professional development (Gao et al., 2011). Among diverse professional development choices, research engagement has been strongly suggested as an innovative model for its potential as a powerful transformative force in English as a foreign language (EFL) teachers’ work and professional development (Borg, 2010). It is now a requirement for faculty members in research institutions and all types of institutions to publish (Lucas and Murry, 2011), which attracted scholars to study individual-level research productivity and factors that contribute to its increase (Nygaard, 2017). Uwizeye et al. (2021) found that, as individual factors, academic qualifications, gender, motivations, and research self-efficacy had the most consequences on teachers’ research productivity in African higher education institutions. Among those institutional factors, research environments, or cultures, are considered as the most influential ones that impact research productivity (Ajjawi et al., 2018). Similarly, mentoring affects the mentee, mentor, and organization positively (Eby and Robertson, 2020). In order to have a better understanding of teachers’ research productivity, we focus on those aspects relating to teachers’ psychology, namely, research motivation, self-efficacy, and their mediating role in influencing institutional policies on teachers’ research productivity, particularly the provision or absence of mentorship and the working environment of EFL teachers in China.

We propose that the mediating mechanism be used to explain how university EFL teachers’ motivation and self-efficacy as mediators affect their research productivity. To this end, we have three specific aims: (1) to investigate the mediating role of teachers’ research motivation and self-efficacy in the relationship between institutional support and research productivity, (2) to broaden the former studies which focused on the direct effect on research productivity, (3) to provide practical information, especially for administrators in higher education to increase teachers’ research productivity.

## Literature Review

Many studies have been conducted to identify factors that affect an individual’s research productivity (Freedenthal et al., 2008). These factors were categorized into individual and institutional factors (Uwizeye et al., 2021). Individual factors included teachers’ motivation and self-efficacy, while institutional factors included mentorship and working environment. Following this line of research, we introduced teachers’ mentorship and working environment mediated by their research motivation and self-efficacy as a mechanism of affecting research productivity.

### Research Productivity

The appearance of the construct of research productivity could date back to the early 1970s (Creswell, 1985), and it is defined as the number of publications in academic refereed journals and/or scholarly books as well as presentations in conferences which usually have the chief function of productivity measure for promotion and tenure in university (Dundar and Lewis, 1998). However, different institutions and disciplines vary in measuring productivity (McGill and Settle, 2012; Paul and Mukhopadhyay, 2022). Generally, for university teachers, the number of research publications in top-ranked journals over the past 10 years (Long et al., 2009), reports, monographs, book chapters, book reviews, books, and the amount of research funding awarded are often used to assess their research productivity (Blackburn and Lawrence, 1995). Despite research productivity being crucial for teachers and universities, studies on research productivity amongst university teachers and sole and joint productivity analysis in academia are rather limited (Jang and Shin, 2011). Borg (2007) has also called for empirical research on the EFL teachers’ research engagement. Additionally, Heng et al. (2022) reported that the academics’ research engagement was limited in the marginalized global south nations, as well as countries such as China, and such a situation exacerbated during the pandemic. Therefore, they appealed that research be conducted to better understand how the fragile research environment would affect teachers’ research, and enhance their research productivity.

### Institutional Support and Research Productivity

Research support is defined as any provided resource that can boost a faculty member’s ability to engage in scholarship (McGill and Settle, 2012). Previous research has investigated the main types of institutional support and the relationship between institutional support and research productivity (Freedenthal et al., 2008). According to these studies, institutional support includes three sub-constructs: research mentoring experience (i.e., being mentored in research), research environment, and research education (Jang and Shin, 2011). Among various support, mentorship is the most prominent factor affecting teachers’ research productivity (Allen et al., 2018). And a beneficial working environment bolsters teachers’ research productivity (Aboagye et al., 2021). Luo and Hyland (2016) have found that a lack of institutional support is one of the main reasons why Chinese university teachers’ manuscripts cannot be published and even if they get published, their work is cited less frequently.

### Mentorship Support

Mentorship is a kind of institutional support in which a more-experienced member supplies information, support, and guidance to a less-experienced, usually new member of an institution to promote the successful chances of the latter within or beyond the institution (Campbell and Campbell, 1997). Transferring skills and supporting continuous learning, especially when skills are scarce, are the main functions of mentoring (Nundulall and Dorasamy, 2010). General guidance and skill development training from the relevant technical expertise also enhance research engagement in university teachers (Wilkins, 2011). For instance, Loewen (2019) reported that language teachers are neither paid nor trained to do research. In his meta-analysis of 43 studies on mentoring, Simmering (2007) found that teachers’ research output is comparatively low with a lack of mentorship programs. Likewise, mentorship programs will increase research output (Nundulall and Reddy, 2011). Besides, studies have found that the availability of training facilities at universities can improve the publication rates of university teachers (Phillips and Russell, 1994). Similarly, Kelly and Warmbrod (1986) found that the lack of training and reflection of research hindered university teachers’ research productivity. In addition, engagement in context-sensitive activities and networking opportunities is vital for university teachers because this can help them secure internal and external funding and be exposed to suitable methods for publishing books and articles (Shaw, 2002).

### Working Environment Support

Research environment refers to the behaviors that include, at a minimum, shared values, assumptions, beliefs, rituals, and the valued, worthwhile, and pre-eminent activity with a central focus on the acceptance and recognition of research practices and outcomes (Evans, 2007). It is found that faculty’s work environments drove their productivity and prominence (Way et al., 2019). It was necessary for higher education institutions to provide a conducive research environment for academics to stimulate their engagement with research (Tadesse and Khalid, 2022). In reality, studies found that teachers had unsatisfied work environments. With limited available time for research, teachers were also imposed by the excessive workload that results in their lower research productivity as well as fewer opportunities for research training (Griffiths et al., 2010). Similarly, Kelly and Warmbrod’s (1986) qualitative research revealed that heavy teaching load had a negative impact on the university teachers’ research productivity. In China, the limited educational resources were adverse to language teachers’ professional development (Gao and Xu, 2014).

Across various contexts and disciplines, lack of time acted as a negative mechanism leading to decreased research productivity (Ajjawi et al., 2018). Because of that, some researchers emphasized the necessity of separating research from teaching hours in faculty time allocation (Creswell, 1985), and they argued that universities must arrange schedules that allowed teachers to have sufficient time to gather resources and conduct research (Graves et al., 1982). Besides time support, adequate financial support can positively affect teachers’ research productivity (Jung, 2012). The allocation of funding for research was output-driven, usually in the form of academic publications (Nundulall and Dorasamy, 2010). Realizing that, department heads and chairs have provided institutional support to increase teachers’ research engagement, such as supporting research travel and nominating teachers for research honors and awards, as Bland et al. (2005) reported. In a study conducted by Dundar and Lewis (1998) in the United States, it was found that funding-related support and faculty productivity had a positive relationship. Increasing institutional funding for teachers would improve teachers’ research productivity (McGill and Settle, 2012). Also, McGill and Settle (2012) discovered that teachers who received more institutional funding were more likely to engage in research. More studies still need to be conducted to explore how institutions can better support university teachers’ research quality and productivity (Dundar and Lewis, 1998).

### Teachers’ Research Motivation and Research Productivity

It is found in recent empirical studies that psychological factors are valuable in explaining research productivity (Hemmings and Kay, 2016). Among these factors, motivation is a prominent one. The definition of motivation is “the dynamically changing cumulative arousal in a person that initiates, directs, coordinates, amplifies, terminates, and evaluates the cognitive and motor processes whereby initial wishes and desires are selected, prioritized, operationalized, and (successfully or unsuccessfully) acted out” (Dörnyei and Ottó, 1998, p. 65). Motivation can be divided into intrinsic motivation and extrinsic motivation: intrinsic motivation refers to the internal fascination and gratification of the activity itself as the main reasons to attract people to engage in an activity, while extrinsic motivation means incentives or external pressures that attract people to pursue an activity (Reeve, 1995).

#### Extrinsic Motivation

As for external incentives, substantial incentives have been used to facilitate teachers to do research. Substantial incentives such as income increases and bonuses took a more significant role than non-substance encouragement, such as the certification and honorary title award in Chinese universities (Henley and Nyaw, 1986). For instance, Santo et al. (2009), who investigated the faculty at the School of Education in Midwestern America, found that the department’s lack of financial support limits the teachers’ opportunities to attend academic-related activities, thus leading to low research productivity among them. Meanwhile, in another study, Creamer (1998) identified department heads or deans who considered research and research productivity the center of rewards. More specifically, Brewer and Brewer (1990) found that 42 of the responding deans in their sample believed that the presence of a merit pay system could and/or do increase faculty research productivity. Besides the financial rewards, it is found that teachers had moderately different patterns of research productivity with varying statuses of tenure (McNurlen and West, 2000). However, the findings are opposite, involving no relationship, negative relationship, and positive relationship between teachers’ tenure and research productivity (Chen et al., 2010). For example, Teodorescu (2000) found no effect of tenure on research productivity. Inversely, Chen et al. (2010) noted that the relationship between research productivity and tenure was strong so that universities took great advantage of it to make teachers’ research productive. Whereas Santo et al. (2009) found tenure and research productivity had a negative relationship because teachers worried nothing about obtaining tenure, thus their research motivation to publish decreased. Especially for those tenured teachers, who may be in a semi-retired state but still employed by the university, they hardly had the motivation to do research (Chen et al., 2006). Besides tenure, the promotion also affects teachers’ research.

From the management perspective, the promotion has been considered as one of the effective ways of encouraging productivity among university teachers (Lai, 1990). Outstanding external rewards make faculty members try their best when promotion and tenure decisions are forthcoming, while less effort after promotion; foretelling fluctuations in productivity through time (Hu and Gill, 2000). However, after achieving the title of full professor and as retiring faculty member, the research productivity is ultimately a recession in their later academic life (Hu and Gill, 2000). Realizing this, universities have been using promotion as an extrinsic motivational tool to boost the research output of university teachers (Chen et al., 2010). Meanwhile, as research output is one of the most important indicators in academic promotion assessment, the promotion has been a robust extrinsic motivator on research productivity (Chen et al., 2006). Similarly, Tien and Blackburn (1996) found that the expected research productivity remains low because of no conferred promotion reward. Higher education institutions could influence academic staff’s research behavior by manipulating the reward structure for promotion (Fox, 1985).

Generally, most universities have specific and clear performance appraisal documents for teachers. However, the workload and research productivity requirements of teachers differ from one university to another. For example, teachers in Chinese higher education institutions are evaluated on their teaching, research, administration, curriculum, and subject construction (Liu and Yu, 2013). To improve the quantity and quality of research, almost every university has *Research and Teaching Office*, which is a traditional department for different levels educational authorities in China to administrate research and pedagogical innovation activities (Gao et al., 2010). Teachers will be in different research-engaged statuses in various institutions because of the different policies on appraising publications. Meanwhile, teachers’ salary depends on their professional titles in combination with their professional performance, in which research productivity accounts for a large proportion (Luthans and Stajkovic, 1999). Additionally, Borg (2009) proposed that English teachers were mainly driven by practical (e.g., solving teaching problems, identifying better teaching ways) and professional (e.g., professional development) concerns to conduct research.

#### Intrinsic Motivation

Besides extrinsic motivation, intrinsic motivation also plays a vital role in motivating teachers to do research. McKeachie (1982) has postulated that academics publish for the enjoyment of the process of inquiry rather than the external rewards. Differently, while expressing their own willingness to integrate research into teaching, teachers are dismissive of other teachers who lack interest in research (Sato and Loewen, 2019). The sense of satisfaction about discovery, such as defining research goals and outlining paths to achievements, gives academics satisfying emotions, leading them to high work motivation (Stark, 1986). Moreover, intrinsic motivation is significant in the preference for autonomy and independence as well as achieving something on one’s own (Blackmore and Kandiko, 2011). Thus, intrinsic motivation creates teachers engaging in research actively (Trembley et al., 2009). However, teachers with research experience and skills are sometimes demotivated by their perception of the discrepancy between their preferred research and the institutions that encourage them to conduct (Kyaw, 2021).

### Teachers’ Research Self-Efficacy and Research Productivity

Research self-efficacy is an individual’s beliefs about his or her ability to carry out research (Morrison and Lent, 2014). Generally, studies on teachers’ research self-efficacy are divided into three categories. First, previous studies have shown that research self-efficacy has a positive relationship with research disposition which consists of research interest and research experience (Bandura and Adams, 1977; Bard et al., 2000; West et al., 2007). Specifically, research interest has a high association with research self-efficacy (Bard et al., 2000; West et al., 2007) and research experience will bolster research self-efficacy (Love et al., 2007). Second, research self-efficacy correlates with research support directly (Jang and Shin, 2011). Research support involves three sub-constructs: research mentoring experience (i.e., being mentored in research), research training environment, and research education (Jang and Shin, 2011). Specifically, research mentoring experience associates with research self-efficacy positively (Hollingsworth and Fassinger, 2002) as well as research training environment and research education (Holland, 1985; Judge et al., 2007). Third, research self-efficacy will impact research outcome. Research outcome refers to research outcome expectation (Bard et al., 2000) and research productivity (Phillips and Russell, 1994; Kahn and Scott, 1997). Specifically, Bieschke et al. (1998) found there is a strong positive relationship between research self-efficacy and research outcome expectation. However, the correlation between teachers’ self-efficacy and research productivity has inconsistent findings. For example, Landino and Owen (1988), Vasil (1992), and Kahn and Scott (1997) have found that self-efficacy correlates positively with university teachers’ research productivity, inversely, Pasupathy and Oginga (2014) found that the correlation between research self-efficacy and research productivity is weak. Chinses scientific research fails to establish an international reputation (Lin and Fan, 1990) as their limited multilingual capabilities in scholarly publication (Zheng and Gao, 2016). More recently, studies have shown that teachers’ ability to do research is closely linked to their professional identities (Yuan and Zhang, 2020). From the given literature, it can be deduced that the influence of research self-efficacy on research outcome should be further studied to provide more implications for higher education (Jang and Shin, 2011). Besides, the relationship between self-efficacy and academic achievement has been widely studied in the context of the tertiary English as second language (ESL) (i.e., contexts where English is the dominant or first language in education, law and every sphere in society and those who learn it in such native-speaking contexts are ESL learners and users). However, such studies have been scarcely reported in relation to an EFL context (Noorollahi, 2021). To fill this gap, our study investigated how the academics were affected by their self-efficacy beliefs in China, a typical EFL context, where English is seldom used in society as a working language; nor is it even used informally for daily communication. English is offered as a subject in schools and universities and taught as a foreign language (Zhang, 2021b).

### Meditation Effect of Motivation and Self-Efficacy on Research Productivity

A supportive research environment could affect teachers’ intrinsic motivation in research and their productivity (Peng and Gao, 2019); also, research self-efficacy correlates with research support directly (Jang and Shin, 2011). However, few studies have examined the interaction between individual and institutional characteristics and how individuals handle conflicting goals or priorities (Nygaard, 2017). Academics’ priority is affected by their self-efficacy and the perception of organizational priorities (Williams and Kotrlik, 2004). Findings from recent studies imply that there might be mediation or moderation between research productivity and the research environment by diverse organizational and individual-level factors (Ajjawi et al., 2018). For example, Gelso and Lent (2000) indicated that the self-efficacy plays a mediation role between factors, such as research training environments and ultimate continuum outcomes (e.g., research productivity). However, empirical research into the potential mediational role between institutional factors and research productivity is scarce (Kozhakhmet et al., 2022). Specifically, it is necessary to examine the mediating function of individual characteristics between institutional factors and research productivity (Holttum and Goble, 2006). Based on the above considerations, the following hypotheses are proposed:

H1: Both motivation and self-efficacy mediate institutional support and research productivity.
H2: Either motivation or self-efficacy mediates institutional support and research productivity.
H3: Neither motivation nor self-efficacy mediates institutional support and research productivity.

## Methodology

### Participants

The data of this study were gathered through a questionnaire survey sent to Chinese university EFL teachers. As long as they were teaching English at the university level, they were eligible to participate in this survey. The snowball sampling method (Dörnyei, 2007) was adopted in this study for it was the most comprehensive way to collect the representative data in China. With the snowball sampling method, participants who met the criteria of the present study were contacted first then, these teachers were asked to pass on the information to other teachers who might be interested in participating in this study (Dörnyei and Taguchi, 2010). Finally, a total of 536 teachers responded to the questionnaire.

### Instrument

The inventory of *Questionnaire on Teachers’ Research Productivity (QTRP)* was composed of four subscales respectively named *Questionnaire on Teacher Research Self-efficacy (QTRSE), Questionnaire on Teacher Research Motivation (QTRM)*, and *Questionnaire on Institutional Support for Teacher Research (QISTR)* with 37 items in total to test the influence of these factors on teachers’ research productivity as well as a one-factor scale with three items measuring teachers’ research productivity (*Questionnaire on Teachers’ Research Productivity*).

Teachers chose the degree level of agreeing with each item on a six-point rating scale. The scale had three negative responses and three positive responses symmetrically, 1 (Strongly Disagree), 2 (Disagree), 3 (Moderately Disagree), 4 (Moderately Agree), 5 (Agree), 6 (Strongly Agree). Teachers were asked to tick the response box corresponding to their beliefs about each item.

### Procedures for Data Collection

An invitation email was sent to the heads of school to get their permission for disseminating the questionnaire to their faculty during the first semester in 2019–2020. After the head approved of the researcher’s request, an invitation to respond to the questionnaire and the PISs and CIF were delivered to these teachers by the faculty secretary. All the teachers who participated in the survey were told that their responses were confidential.

The participants were requested to specify the numbers of their academic publications in the preceding 10 years (2010–2019) within three categories: (i) scholarly articles in journals, (scholarly articles in journals, specify the nuapplied projects. The manuscripts that are currently in preparation are excluded from counting toward their total research productivity, as they are not complete scholarly work (Wester et al., 2019). Finally, 536 teachers responded to the questionnaire, among which 508 complete questionnaires were taken as valid and were analyzed.

### Analysis

There were two stages in combining the exploratory and confirmatory procedures (Anderson and Gerbing, 1988). Exploratory Factor Analysis (EFA) was used in the self-development scale *Questionnaire on Institutional Support for Teacher Research* to examine the reliability and validation of the construct. The measurement model was determined using EFA by performing maximum likelihood extraction and oblique rotation (Costello and Osborne, 2005). After that, Confirmatory Factor Analysis (CFA) was applied in these four scales: *Teacher Research Self-efficacy*, *Questionnaire on Teacher Research Motivation*, *Questionnaire on Institutional Support for Teacher Research*, and *Questionnaire on Teachers’ Research Productivity* to evaluate the factorial and construct validity for each scale within the measurement model. The total sample (546) was randomly divided into two equal halves with one half being used for exploratory factor analysis (EFA; *n* = 273) and the other half for confirmatory factor analysis (CFA; *n* = 273). The EFA and CFA were performed by SPSS 27 and AMOS 27 separately. As recommended by the previous researchers, a model did not need to be rejected if the following conditions were satisfied (Hu and Bentler, 1999; Fan and Sivo, 2005):

1) χ2 per degree of freedom was statistically non-significant (i.e., χ2/*df* ≤ 3.83),
2) comparative fit index (CFI) and Gamma hat > 0.90,
3) Root mean square error of approximation (RMSEA) < 0.08, with 90% confidence interval being less than 0.08, and
4) Standardized root mean residual (SRMR) < 0.08.
5) The bootstrapping technique was used to test the mediation effect of motivation and self-efficacy.

**FIGURE 1:**
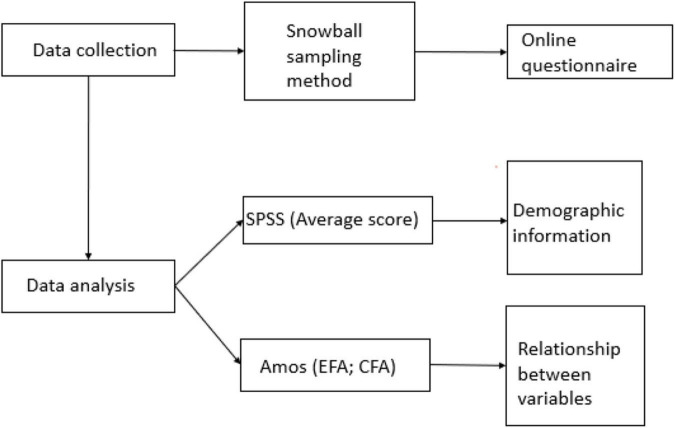
Data collection and analysis procedures.

The concrete data collection and analysis procedures are displayed in Figure 1.

## Results

This section presents the demographic information of the participants, the results of instrument validation and findings about EFL teachers’ research productivity. It also shows how research productivity was affected by the working environment both directly and indirectly, mediated by teachers’ intrinsic motivation, extrinsic motivation, and self-efficacy.

### Demographic Information of the Participants

This study recruited a total of 536 Chinese EFL teachers on a voluntary basis. As expected, more females (380, 74.8%) than males (128, 25.2%) took part in the survey. This is a reflection of the EFL teacher composition in the Chinese education system, where there were more female teachers than male teachers. The age group of teachers between 31 and 40 (38.8%) almost equaled teachers between 41 and 50 (39.2% of the total 536 teachers), constituting 80% of the participants, then the remaining 13.2% and 8.9% of the respondents formed by those under 30 or over 51 separately. Lecturers (226, 44.5%) and associate professors (195, 38.4%) accounted for almost four-fifths of the total 536 participants, and assistant lecturers took up only a tiny percentage, which was twice as many as the full professors in this study. Table 1 displays the specific participant information.

**TABLE 1:** Participant information.

Demographic characteristic	N	Valid%
**Gender**		
Female	380	74.8
Male	128	25.2
Missing	0	-
**Age**		
≤ 30	67	13.2
31–40	197	38.8
41–50	199	39.2
≥51	45	8.9
Missing	0	-
**Rank**		
Assistant lecturer	59	11.6
Lecturer	226	44.5
Associate professor	195	38.4
Full professor	28	5.5
Missing	0	-
Total	508	-

### Measurement Models of Variables

#### Questionnaire on Institutional Support for Teacher Research

Descriptive statistics showed that the average mean scores of these 16 items ranged from 2.71 (SD = 1.60) to 3.79 (SD = 1.39). The skewness and kurtosis indices were between the cutoff value of | 3.0| and | 8.0| separately, indicating the normal distribution for the exploratory analysis (Kline, 2016). Supplementary Appendix 1 shows the descriptive analysis of the *Questionnaire on Institutional Support for Teacher Research* with 16 items.

To conduction exploratory factor analysis (EFA), sampling adequacy was verified with Kaiser-Meyer-Olin by KMO = 0.956. Bartlett’s test of sphericity (*df* = 120, *p* < 0.001) indicates that correlations between items were sufficiently large for an EFA. Maximum likelihood (ML) estimation was employed on the 16 items *via* oblique rotation with Kaiser Normalization, which analyzed the underlying factors that were assumed to be correlated (Field, 2019). The parallel analysis was used to retain components and evaluate the internal reliability of this questionnaire. No factor was removed in this stage. Two predominant factors with more than three indicators each were extracted, explaining 73.62% of the variance.

The two factors were labeled as Factor 1 Mentorship (63.77% variance); Factor 2 Working Environment (9.84% variance). Cronbach’s alpha coefficient for the two factors ranged from 0.944 for Factor 2 to 0.949 for Factor 1. The internal consistency for the two factors met the benchmark value for satisfactory reliability (≥0.70), supporting the significant indicator-construct relationship of the instrument. Table 2 shows the factor loadings and the internal reliability of the two-factor scale.

**TABLE 2:** Factor loadings for exploratory factor analysis and internal reliability of the two institutional supports (*n* = 508).

Factor loading				
	
Factor	Item	1	2	α
Mentorship (M)	M1-item 1	0.482		
	M2-item 2	0.785		
	M3-item 3	0.953		
	M4-item 4	0.955		
	M5-item 5	0.949		0.949
	M6-item 6	0.845		
	M7-item 7	0.643		
	M8-item 8	0.536		
Working Environment (WE)	WE1-item 9		0.626	
	WE2-item 10		0.548	
	WE3-item 11		0.790	
	WE4-item 12		0.832	
	WE5-Item 13		0.817	0.944
	WE6-Item 14		0.923	
	WE7-Item 15		0.893	
	WE8-Item 16		0.854	

*Items with factor loading of 0.30 or greater are included; α = Cronbach’s alpha.*

**FIGURE 2:**
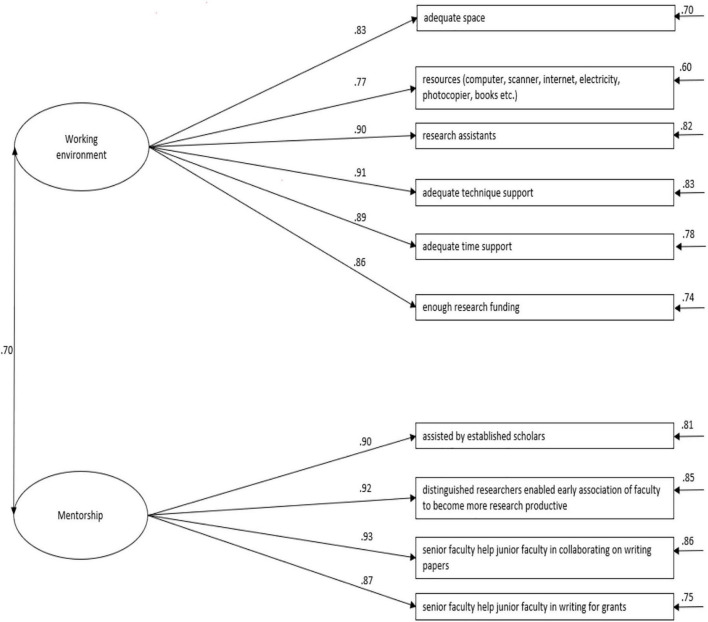
The two-factor correlated model of institutional support on teacher research. Mentorship, mentorship support; working environment, working environment of teachers.

Confirmatory factor analysis (CFA) was used to test the factor structure. The two-factor structure generated in EFA with maximum likelihood (ML) estimator was examined by CFA. A correlated model was constructed on the basis of the EFA results. To improve the modification indices (MI), some adjustments were made to the original factorial structure in turn. By doing so, six items were deleted. These deleted items were shown as follows: Item 1: In my department I have been, or had been, formally assigned an advisor or mentor to help me in research; Item 2: In my department mentors provide emotional and professional support to junior faculty in times of need; Item 7: My department provides many ways for junior teachers to communicate with experienced scholars; Item 8: My department provides training for me to get skills and knowledge to do research; Item 9: My department provides latest literature for me to do research; Item 10: My department provides access to external research resources for me to conduct my research. After the removal of the factors, the final model with acceptable fit indices was produced (see Table 3). Figure 2 illustrates the factor structures of the above-tested models. Table 4 shows regression weights of the two-factor correlated model of institutional support. The mean, standard deviation, effect size for the difference in means, and inter-correlation between the two factors in the final IS Model (Institutional Support Model) are given in Table 5. As depicted in Table 5 and Figure 2, the correlation matrix showed that the two factors were significantly correlated with strong degrees in a positive direction. The satisfactory levels of correlations verified that these factors were distinct enough but also under the same theoretical construct of institutional support, confirming the discriminant validity.

**TABLE 3:** Model fit indices of institutional support model.

Model	Description	χ 2 (*df)*	χ 2/*df*	CFI	Gamma hat	RMSEA	90% CI	SRMR	AIC
IS	IS model (Two-factor, 10 items)	130.735 (34)	3.845***	0.98	0.96	0.075	0.062–0.089	0.047	172.74

***Means the result is significant because it is within the range of 2 ≤ χ2/df ≤ 5.

**TABLE 4:** CFA regression weights for the two-factor correlated model of institutional support.

Institutional support	Unstandardized estimate	Standardized estimate	C.R.
Item3-M1	1.000*^a^*	0.900	a
Item4-M2	0.966	0.920	33.259***
Item5-M3	0.989	0.926	33.839***
Item6-M4	0.927	0.867	28.831***
Item11-WE1	1.000*^a^*	0.834	a
Item12-WE2	0.983	0.772	20.724***
Item13-WE3	1.117	0.904	26.771***
Item14-WE4	1.128	0.909	27.014***
Item15-WE5	1.076	0.886	25.815***
Item16-WE6	1.037	0.862	24.629***

***p < 0.001; “a” means the regression weight was fixed at 1.00 for model identification purposes; hence no critical ratio was computed. M, mentorship; WE, working environment.

**TABLE 5:** Inter-correlation of the two-factor institutional support.

	Inter-correlation				
	
Factor	1	2	M	SD	Effect size
1. Mentorship	1		3.42	1.42	0.17
2. Working environment	0.699	1	3.18	1.36	

The results of EFA and CFA supplied substantial evidence for the factorial structure of the questionnaire, involving mentorship and working environment. The medium strength of the correlations between the two constructs was distinguished but correlated under the same construct of institutional support.

##### Mentorship

The first dimension was defined as mentorship. In this study, the mentorship was measured by four items (e.g., In my department senior faculty help junior faculty in collaborating on writing papers; In my department senior faculty help junior faculty in writing for grants.) for different aspects of mentorship. However, mentorship was paid insufficient attention in the study of the effect of institutional support on research. Therefore, the exploration of the relationship between mentorship and research engagement was in need. Hopefully, the findings of this study would add up more evidence on the relationship of the two factors.

##### Working Environment

The second dimension, labeled as the working environment, refers to provided time, funding, technical expertise, and assistance support from the institution. In this study, the working environment of the EFL teachers in China was investigated through six items (e.g., My department provides resources such as the computer, scanner, internet, electricity, photocopier, books, etc.) for me to conduct my research; My department provides enough research funding to do research). Previous literature has found that working environments affected research productivity of university teachers (Wilkins, 2011). Therefore, this empirical exploration of EFL teachers’ working environment was expected to offer insight into how to provide effective support for improving EFL teachers’ research productivity.

#### Questionnaire on Teacher Research Self-Efficacy

The *Questionnaire on Teacher Research Self-efficacy (QTRSE)* was adapted from *The Self-Efficacy in Research Measure (SERM)* (Phillips and Russell, 1994) to investigate three types of research self-efficacy. As shown in previous literature, the *SERM* has been widely validated in many research settings with sound psychometric properties, this study directly applied CFA to evaluate the validity of the modified instrument *QTRSE* in EFL environments.

Results of confirmatory factor analysis (CFA) produced interesting findings. Based on the previous literature and the theoretical framework of the *SERM*, we hypothesized a three-factor structure as designed in the *SERM* in general academic contexts, which eliminated the Quantitative and Computer Skills factor from the original questionnaire because both quantitative and qualitative skills were employed widely among Chinese EFL teachers, and therefore it unfitted the Chinese context. After that, 10 items were deleted during the preparatory stage as the pilot 60 teachers deemed these items were not suitable in the Chinese context. Finally, there were 15 items loaded onto three factors: Research Design Skills, Practical Research Skills, and Writing Skills. The inspection of modification indices suggested the possibly mis-specified items. The initial model was respecified by removing seven questionable items successively to improve the model fit. Finally, the three-factor model with eight items was defined as the most appropriate research self-efficacy model.

**FIGURE 3:**
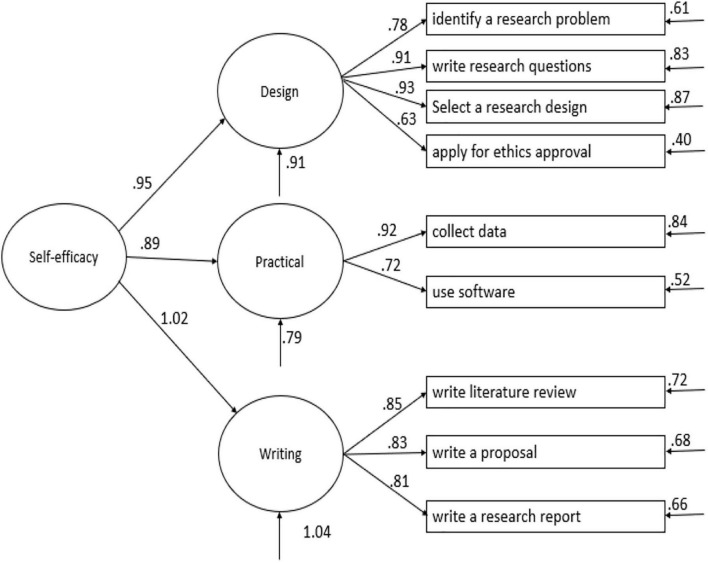
The three-factor correlated model of research self-efficacy. Design, research design skills; practical, practical research skills; writing, writing skills.

Figure 3 shows the correlation model of research self-efficacy. There was a concern with the desired minimum number of three items under each factor in CFA. However, the two-item per factor model could be justified as reliable when the two variables are highly correlated with each other (*r* > 0.70) (Yong and Pearce, 2013). In the present case, the correlation between items W1 and W2 is 0.72, which suggested that the two items could adequately measure the desired factor of writing skills. The factor structures of the above-discussed model are shown in Figure 3. In this correlated model, standardized estimates loadings of all 8-item on the hypothesized latent constructs were higher than 0.70 (see Table 6). Table 7 shows the CFA results of research self-efficacy.

**TABLE 6:** CFA regression weights for the three-factor correlated model of research self-efficacy.

Research engagement	Unstandardized estimate	Standardized estimate	C.R.
Item1-D1	0.960	0.779	23.589***
Item4-D2	1.000*^a^*	0.915	a
Item5-D3	1.010	0.938	36.127***
Item9-P1	1.000*^a^*	0.846	a
Item10-P2	1.084	0.954	30.092***
Item11-P3	1.045	0.921	28.496***
Item3-W1	1.000*^a^*	0.871	a
Item6-W2	0.938	0.823	23.459***

***p < 0.001; “a” means the regression weight was fixed at 1.00 for model identification purpose hence no critical ratio was computed. Design, research design skills; practical, practical research skills; writing, writing skills.

**TABLE 7:** Model fit indices of research self-efficacy model.

Model	Description	χ 2 (*df)*	χ 2/*df*	CFI	Gamma hat	RMSEA	90% CI	SRMR	AIC
RS	RS model (Three-factor, 8 items)	52.221 (17)	3.072***	0.99	0.98	0.064	0.045–0.084	0.018	90.221

CFI, comparative fit index; RMSEA, root mean square error of approximation; SRMR, standardized root mean residual; CI, confidence interval; AIC, akaike information criterion; ***p < 0.001.

The mean, standard deviation, effect size for the difference in means, and inter-correlation between the three factors in research self-efficacy are given in Table 8.

**TABLE 8:** Inter-correlation of the three-factor research self-efficacy.

	Inter-correlation				
	
Factor	1	2	3	M	SD
1. Design	1			3.99	1.03
2. Practical	0.773	1		3.63	1.14
3. Writing	0.956	0.772	1	3.92	1.01
	Effect sizes				
Design compared to			Writing compared to		
Practical	0.33		Practical		
Writing	0.07				0.27

#### Questionnaire on Teacher Research Motivation

Confirmatory factor analysis was used to evaluate the initial seven-factor 22-item *Questionnaire on Teacher Research Motivation*. The examination of the initial model showed the unaccepted model fit indices. To achieve acceptable model fit, three items were removed from the original model in turn. The final well-fitted model rm model was the most parsimony model with 19 items belong to seven factors. Table 9 provides a summary of the model fit indices of the final model of research motivation. Generally, one factor should be measured by at least three items in a scale, as the exception, scales measured more than one factor would be identified with minimum two items per factor (Raubenheimer, 2004), meanwhile, the two items should be highly correlated (*r* > 0.70) (Worthington and Whittaker, 2006). In *QTRM*, the correlation between items SK1 and SK2 is 0.79 while for FL1 and FL2 is 0.77, which implied the two items could adequately measure the desired factor of skills and flexibility. As to AC1 and AC2, the correlation between them is only 0.68, however, this is close to 0.70 showing strong correlation of the two items and arguably acceptable. When speaks the factor loading, if the sample size was over 350, then the factor loading of 0.30 can be acceptable (Yari et al., 2014). Thus, item COM3 (0.37) was accepted in this study.

**TABLE 9:** Model fit indices of research motivation model.

Model	Description	χ 2 (*df)*	χ 2/*df*	CFI	Gamma hat	RMSEA	90% CI	SRMR	AIC
RM	RM model (Seven-factor, 19 items)	505.947 (131)	3.862***	0.95	0.93	0.075	0.068–0.082	0.055	623.947

CFI, comparative fit index; RMSEA, root mean square error of approximation; SRMR, standardized root mean residual; CI, confidence interval; AIC, akaike information criterion; ***p < 0.001.

**FIGURE 4:**
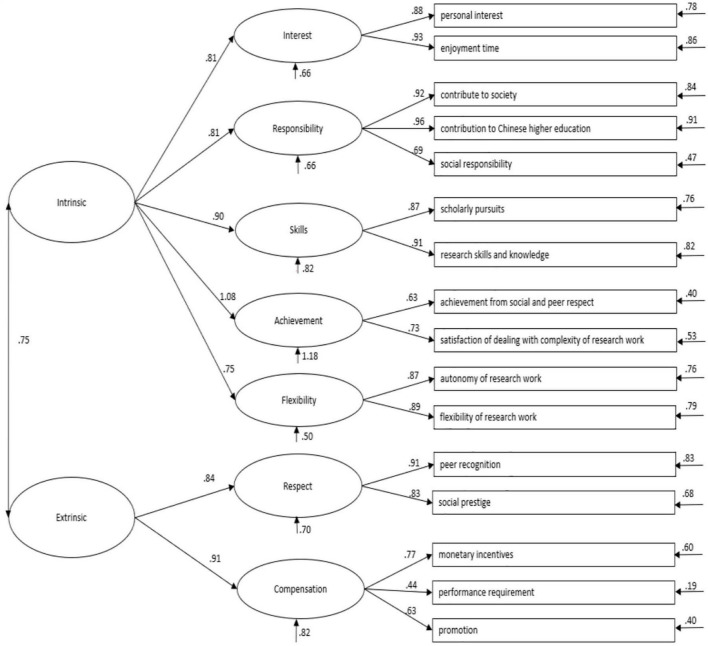
The structure of the seven-factor correlate research motivation model. IN, interest; RE, responsibility; SK, skills; AC, achievement; FL, flexibility; RES, respect; COM, compensation.

Figure 4 shows the structure of the Seven-factor Correlate Model. In this correlated model, standardized estimates loadings of all 19-item on the hypothesized latent constructs were almost higher than 0.50 beside COM3 (0.37) (see Table 10). The mean, standard deviation, effect size for the difference in means, and inter-correlation between the seven factors in research motivation were given in Tables 11, 12.

**TABLE 10:** CFA regression weights for the seven-factor correlated model of research motivation.

Research engagement	Unstandardized estimate	Standardized estimate	C.R.
Item1-IN1	1.000*^a^*	0.880	a
Item2-IN2	1.047	0.916	27.780***
Item3-IN3	0.747	0.751	20.422***
Item8-RE1	1.000*^a^*	0.921	a
Item9-RE2	1.032	0.954	36.450***
Item10-RE3	0.710	0.683	18.913***
Item4-SK1	1.000*^a^*	0.871	a
Item5-SK2	0.955	0.915	20.077***
Item6-AC1	1.000*^a^*	0.850	a
Item7-AC2	0.939	0.803	21.129***
Item13-FL1	1.000*^a^*	0.849	a
Item14-FL2	1.074	0.912	21.548***
Item15-RES1	1.000*^a^*	0.811	a
Item16-RES2	1.189	0.936	25.935***
Item17-RES3	1.141	0.914	25.191***
Item18-COM1	1.000*^a^*	0.788	a
Item19-COM2	0.949	0.686	14.554***
Item20-COM3	0.398	0.371	7.731***
Item22-COM4	0.809	0.548	11.536***

***p < 0.001; “a” means the regression weight was fixed at 1.00 for model identification purpose hence no critical ratio was computed. IN, interest; RE, responsibility; SK, skills; AC, achievement; FL, flexibility; RES, respect; COM, compensation.

**TABLE 11:** Inter-correlation of the seven-factor research motivation.

	Inter-correlation								
	
Factor	1	2	3	4	5	6	7	M	SD
1. Interest	1							4.01	1.28
2. Responsibility	0.662	1						4.17	1.16
3. Skills	0.756	0.758	1					4.49	1.21
4. Achievement	0.651	0.774	0.878	1				4.63	1.15
5. Flexibility	0.628	0.605	0.606	0.604	1			4.17	1.21
6. Respect	0.485	0.603	0.551	0.731	0.606	1		4.15	1.10
7. Compensation	0.473	0.580	0.614	0.728	0.663	0.776	1	4.42	0.97

**TABLE 12:** Effect sizes of the seven-factor research motivation.

Factors	Effect sizes	Factor	Effect sizes	Factor	Effect sizes	Factor	Effect sizes	Factor	Effect sizes
Interest compared to		Responsibility compared to		Skills compared to		Achievement compared to		Flexibility compared to	
Responsibility	−0.13	Skills	−0.27	Achievement	−0.12	Flexibility	0.39	Respect	0.02
Skills	−0.38	Achievement	−0.40	Flexibility	0.26	Respect	0.43	Compensation	−0.23
Achievement	−0.51	Flexibility	0	Respect	0.29	Compensation	0.20		
Flexibility	−0.13	Respect	0.02	Compensation	0.06			Respect compared to	
Respect	−0.12	Compensation	−0.23					Compensation	−0.26
Compensation	−0.36								

#### Questionnaire on Teachers’ Research Productivity

Because research productivity is a single factor-dependent variable, the measurement test (CFA) showed a saturated model, and goodness of fit tests was not available. As a saturated model, it has as many parameters as data points to which it is fitted with zero degrees of freedom (Agresti, 2002). For this kind of model, there was no estimation on model fit indices but focused on only the path coefficient (Steeger and Gondoli, 2013). All of the three observed variables reflected research productivity positively. Projects had the most robust relationship with research productivity (*r* = 0.71, *p* < 0.001), and articles (*r* = 0.67, *p* < 0.001) impacted research productivity relatively lower than projects. However, articles had a much greater influence on research productivity than conferences (*r* = 0.51, *p* < 0.001).

### Structural Model of Direct and Indirect Effect on Research Productivity

Two of the four exogenous variables significantly affected research productivity. Besides, two factors among all the twelve latent variables directly affected research productivity. The working environment influenced research productivity negatively (β = −0.263, *p*<0.05). Inversely, teachers’ research self-efficacy (β = 0.351, *p*<0.05) positively affected research productivity. The research productivity was explained 13.7% (SMC = 0.137, *p*<0.001) in total by this model, and the effect size of this model was 0.159. Table 13 shows the model fit index of the RP Model (Research Productivity Model). Table 14 displays the regression weights.

**TABLE 13:** Results of the Structural Equation Modeling (SEM) for research productivity.

Model	Description	χ 2 (*df)*	χ 2/*df*	CFI	RMSEA	90% CI	TLI	Gamma hat	SRMR
RP	RP model (Four-factor, 40 items)	1553.041 (678)	2.291***	0.95	0.050	0.047–0.054	0.94	0.92	0.0531

***Means the result is significant because it is within the range of 2 ≤ χ2/df ≤ 5.

**TABLE 14:** The statistical value of the effect analysis on research productivity.

Dependent variables	Research productivity		
	
Independent variables	Total effect (TE)	Indirect effect (IE)	Direct effect (DE)
Intrinsic motivation	0.427***	000	0.427***
Extrinsic motivation	−0.253***	000	−0.253***
Self-efficacy	0.217***	000	0.217***
Mentorship	0.130	0.032	0.098
Working environment	–0.098	0.170***	−0.267***

***p < 0.05.

### Mediating Roles of Researchers’ Motivation and Self-Efficacy on Research Productivity

**FIGURE 5:**
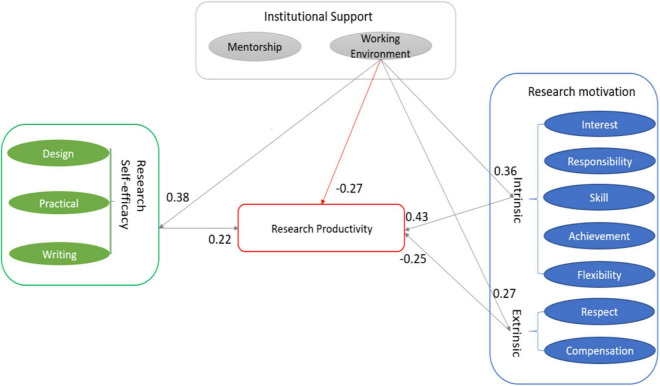
The influence on teachers’ research productivity.

The working environment, teachers’ research motivation, and self-efficacy directly influenced teachers’ research productivity. However, teachers’ extrinsic research motivation negatively influenced teachers’ research productivity in this study. Based on the existing theoretical framework, this study also examined the influence of institutional support on teachers’ research productivity as mediated by teachers’ research motivation and self-efficacy. The result shows that motivation and self-efficacy significantly mediate the relationship between working environment and productivity (β = 0.170, *p*<0.05, 95% CI, 0.097–0.260). The result revealed that the working environment is significantly related to teachers’ research productivity indirectly mediated by motivation and self-efficacy. Figure 5 displays both the direct and indirect influence of teachers’ research motivation, self-efficacy, mentorship, and the working environment on their research productivity.

## Discussion

Research motivation and self-efficacy mediate the indirect relationship between the working environment and research productivity, proving H1. It also added evidence that environmental factors have a powerful influence on individual variables (Lent et al., 2000). The intermediary model shows the following two relationships. Firstly, it is found that teachers’ research motivation plays a vital role in bridging the working environment and research productivity. Notably, Nguyen’s (2021) presented that the perceived institutional support directly affects teachers research motivation, therefore, impacts teachers’ research productivity. Specifically, intrinsic motivation positively mediates this relationship, suggesting that improving the working environment encourages teachers to produce more. It is because the provided convenient environment motivates teachers to engage in research more easily and efficiently. Naturally, the research outcomes would increase. Corroborating the findings of Trembley et al. (2009), enhancing intrinsic motivation leads to increasing research productivity. Inversely, extrinsic motivation plays a negative mediation role. Teachers would decline their research engagement whenever they achieve their goals successfully (Santo et al., 2009) or feel hopeless to fulfill the career objectives. This may be caused by teachers considering the rewards for research never appeal to them. Teachers engage less in research because the inadequate working environment declines their extrinsic motivation for research reducing the research outputs. Another possible explanation is that the insufficient support from the institution makes teachers confront intellectual and financial challenges, and they had to squeeze time to do research (Gao et al., 2010). Those teachers might give up research if they did not internally motivated. Therefore, it can be argued that it is a wise choice for universities to stimulate teachers’ intrinsic motivation to do research rather than the external drive. Second, it is revealed that self-efficacy positively mediates the working environment and research productivity. Corroborating Nguyen’s (2021) findings, the researcher found, with the advanced working environment, teachers are more confident in doing research. Promoting the working environment increases research self-efficacy and then enhances individuals’ research productivity. No matter what and when they need research support, teachers could get smoothly from their department, which would improve their confidence level for research. The confidence level of teachers is in proportion to their research engagement. Undoubtedly, it enhances teachers’ research productivity indirectly.

As regards the direct relationship in this study, the current findings suggest that mentorship is not significantly correlated with teachers’ research productivity corresponding with Simmering’s (2007) finding. That may be because no mentors help teachers publish, and teachers get little help from experienced academics in Chinese higher education institutions. Teachers in Chinese universities are used to doing research alone because of the fiercely competitive relationship among colleagues. The institutional practice of research appraisal gives credit to only the first author, which might be a significant reason that deters Chinese university teachers from collaboration or co-authoring research publications. Hence, mentoring does not have any social role in this case. However, the collaboration of research between scholars within or without the same institutions is a trend worldwide (Paul and Mukhopadhyay, 2022). Therefore, Chinese scholars are inspired to consider cooperating with other scholars in publishing to improve their own rating and the ranking of their institutions. This study also found that the working environment is negatively correlated with teachers’ research productivity. The negative association between working environment and research productivity may imply that when teachers are pressured to publish whereas publication beyond their reach, they might be surrender for having little support from their department. Compared with other universities, the universities in question provide inadequate support for teachers to do research. Firstly, these teachers had to squeeze time to do research and pay money for publishing by themselves. Secondly, the research fundings were limited, and the number of applicants was far more than the projects. At last, the sources of research, such as online databases, conferences, workshops, etc., are insufficient in China. Teachers struggle for publishing when they have to work alone, which restricts their research productivity as seen from the number of research outputs.

## Conclusion and Implications

This study examined individual factors as the mediating mechanism, by which institutional factors affected teachers’ research productivity. First of all, it was indicated that the working environment was negatively associated with their productivity. Mentorship had no relationship with teachers’ research productivity. Second, it was revealed that teachers’ research motivation was crucial in bridging working environment and research productivity; similarly, teachers’ research self-efficacy had the same mediating function. Hence, the result supported hypothesis 1 that motivation and self-efficacy mediated the working environment and research productivity. Therefore, a supportive work environment where teachers feel comfortable doing research multiplies the effect of self-efficacy and intrinsic motivation on teachers’ research productivity. In efforts to improve teachers’ research productivity, higher education institutions need to provide adequate institutional support to these university teachers. It is crucial for teachers working in the Chinese context, where they have very few publications and are therefore less frequently cited (Luo and Hyland, 2016), to enhance their work environments to improve research productivity. Teachers gradually increase their confidence in doing research from the improved research environment, which would promote their enthusiasm for research. Institutions would also benefit from providing an appropriate research environment to teachers to increase their research productivity (Gao et al., 2010). This serves another purpose, namely, to boost the institutional ranking of their universities. This means that administrators should offer different support according to the practical situations of their institutions.

Despite the interesting findings, we need to point out the limitations of this study. First, the participants in our study are Chinese EFL teachers, which means that our sample does not represent other groups of teachers. The findings should also be interpreted with this limitation in mind. We encourage colleagues who are interested in the findings to replicate this study in other disciplines or in different contexts. Second, research support in various universities in China is different, and we need to state that these differences could not be reflected through the questionnaire. Future studies might need to use additional techniques for collecting the data to investigate how these differences are played out. Possible tools could include interviews, observation, or other qualitative methods that describe the different types of research support for teachers in detail and depth. Third, this study only clarifies the mediated function of motivation and self-efficacy. Further studies are needed to examine the mediation role of other individual factors.

## Data Availability Statement

The raw data supporting the conclusions of this article will be made available by the authors, without undue reservation.

## Ethics Statement

The studies involving human participants were reviewed and approved by The University of Auckland Ethics Committee on Human Participants. The patients/participants provided their written informed consent to participate in this study.

## Author Contributions

YL designed this study, collected the data, and wrote the first draft. LZ contributed to the interpretation of results and revision of the subsequent versions of the manuscript prior to its submission as the corresponding author. Both authors contributed to the article and approved the submitted version.

## Conflict of Interest

The authors declare that the research was conducted in the absence of any commercial or financial relationships that could be construed as a potential conflict of interest.

## Publisher’s Note

All claims expressed in this article are solely those of the authors and do not necessarily represent those of their affiliated organizations, or those of the publisher, the editors and the reviewers. Any product that may be evaluated in this article, or claim that may be made by its manufacturer, is not guaranteed or endorsed by the publisher.
