# Supplementary material for: Influence of Mentorship and the Working Environment on English as a Foreign Language Teachers’ Research Productivity: The Mediation Role of Research Motivation and Self-Efficacy
Source: Front Psychol. 2022 Jun 15;13:906932. doi: 10.3389/fpsyg.2022.906932 (PMC9240233; doi:10.3389/fpsyg.2022.906932)
Supplement: Supplementary file 1 [file Data_Sheet_1.docx]

**Appendix 1**

**Descriptive Statistics of the *Questionnaire on Institutional Support for Teacher Research***

| Scales and Items | *M* | *SD* | Skewness | Kurtosis |
| --- | --- | --- | --- | --- |
| Institutional Support |  |  |  |  |
| Mentorship (M) |  |  |  |  |
| M1 | 2.71 | 1.60 | 0.53 | -0.92 |
| M2 | 3.18 | 1.57 | 0.06 | -1.15 |
| M3 | 3.35 | 1.59 | -0.05 | -1.11 |
| M4 | 3.60 | 1.50 | -0.24 | -0.91 |
| M5 | 3.43 | 1.52 | -0.11 | -1.00 |
| M6 | 3.30 | 1.52 | -0.02 | -1.03 |
| M7 | 3.51 | 1.48 | -0.17 | -0.87 |
| M8 | 3.79 | 1.39 | -0.38 | -0.56 |
| Working Environment (WE) |  |  |  |  |
| WE1 | 3.62 | 1.48 | -0.20 | -0.82 |
| WE2 | 3.78 | 1.45 | -0.31 | -0.70 |
| WE3 | 3.44 | 1.50 | -0.10 | -0.86 |
| WE4 | 3.49 | 1.59 | -0.06 | -1.05 |
| WE5 | 2.90 | 1.54 | 0.38 | -0.92 |
| WE6 | 3.17 | 1.55 | 0.19 | -0.98 |
| WE7 | 3.05 | 1.52 | 0.29 | -0.86 |
| WE8 | 3.05 | 1.50 | 0.31 | -0.82 |
| Valid N (listwise) | 508 |  |  |  |
